# Electroconvulsive therapy-induced brain functional connectivity predicts therapeutic efficacy in patients with schizophrenia: a multivariate pattern recognition study

**DOI:** 10.1038/s41537-017-0023-7

**Published:** 2017-05-11

**Authors:** Peng Li, Ri-xing Jing, Rong-jiang Zhao, Zeng-bo Ding, Le Shi, Hong-qiang Sun, Xiao Lin, Teng-teng Fan, Wen-tian Dong, Yong Fan, Lin Lu

**Affiliations:** 1 Institute of Mental Health, National Clinical Research Center for Mental Disorders, Key Laboratory of Mental Health and Peking University Sixth Hospital, Peking University, Beijing, 100191 China; 2 National Laboratory of Pattern Recognition, Institute of Automation, Chinese Academy of Sciences, Beijing, 100190 China; 3 University of Chinese Academy of Sciences, Beijing, 100049 China; 4 Department of Alcohol and Drug Dependence, Beijing Hui-Long-Guan Hospital, Peking University, Beijing, 100096 China; 5 National Institute on Drug Dependence and Beijing Key laboratory of Drug Dependence, Peking University, Beijing, 100191 China; 6 Peking-Tsinghua Center for Life Sciences and PKU-IDG/McGovern Institute for Brain Research, Peking University, Beijing, 100871 China; 7 Department of Radiology, Perelman School of Medicine, University of Pennsylvania, Philadelphia, PA 19104 USA

## Abstract

Previous studies suggested that electroconvulsive therapy can influence regional metabolism and dopamine signaling, thereby alleviating symptoms of schizophrenia. It remains unclear what patients may benefit more from the treatment. The present study sought to identify biomarkers that predict the electroconvulsive therapy response in individual patients. Thirty-four schizophrenia patients and 34 controls were included in this study. Patients were scanned prior to treatment and after 6 weeks of treatment with antipsychotics only (*n* = 16) or a combination of antipsychotics and electroconvulsive therapy (*n* = 13). Subject-specific intrinsic connectivity networks were computed for each subject using a group information-guided independent component analysis technique. Classifiers were built to distinguish patients from controls and quantify brain states based on intrinsic connectivity networks. A general linear model was built on the classification scores of first scan (referred to as baseline classification scores) to predict treatment response. Classifiers built on the default mode network, the temporal lobe network, the language network, the corticostriatal network, the frontal-parietal network, and the cerebellum achieved a cross-validated classification accuracy of 83.82%, with specificity of 91.18% and sensitivity of 76.47%. After the electroconvulsive therapy, psychosis symptoms of the patients were relieved and classification scores of the patients were decreased. Moreover, the baseline classification scores were predictive for the treatment outcome. Schizophrenia patients exhibited functional deviations in multiple intrinsic connectivity networks which were able to distinguish patients from healthy controls at an individual level. Patients with lower classification scores prior to treatment had better treatment outcome, indicating that the baseline classification scores before treatment is a good predictor for treatment outcome.

## Electroconvulsive therapy: Connectivity networks reveal good candidates for brain stimulation

Connectivity patterns in the brain may help identify patients with schizophrenia most likely to benefit from electroconvulsive therapy. A team led by Lin Lu from Peking University, China, and Yong Fan from the University of Pennsylvania, USA, took functional magnetic resonance imaging (MRI) scans of 34 people with schizophrenia and 34 control individuals without mental illness. Those with schizophrenia were scanned before and after treatment; some received antipsychotics alone, others received medication plus electroconvulsive therapy. The researchers created organizational brain maps known as “intrinsic connectivity networks” for each individual, and showed that the neuroimaging pattern could discriminate between people with and without schizophrenia. For the schizophrenia patients, the connectivity networks taken prior to treatment also helped predict who would benefit from the brain-stimulation procedure. Such a biomarker could prove a useful diagnostic tool for clinicians.

## Introduction

Schizophrenia is a severe, disabling, and highly heritable psychiatric disorder with an unknown etiology.^1^ Up to 30% of schizophrenia patients do not respond or respond poorly to standard treatment with antipsychotics.^2^ Electroconvulsive therapy (ECT) under general anesthesia with adequate muscle relaxation is an effective treatment for schizophrenia, especially for patients with prominent catatonic symptoms, suicidality, and agitation.^3, 4^ Clinical data have also shown that ECT may serve as an augmentation strategy for treatment-resistant schizophrenia.^4, 5^

Recent studies have demonstrated that ECT in schizophrenia patients alters cerebral blood flow in the prefrontal cortex (PFC),^6^ increases structural network strength in the medial temporal lobe (MTL) network and lateral prefrontal/cingulate cortical network,^7^ increases brain-derived neurotrophic factor (BDNF) levels,^8, 9^ and improves psychotic symptoms. These results suggest that ECT can alter brain connectivity patterns, thereby alleviating symptoms of schizophrenia. However, remaining unclear is the way in which these ECT-induced changes in brain networks are related to the clinical response.

Mounting evidence suggests that resting-state functional connectivity (rsFC) changes reflect clinical states and are correlated with treatment response. For instance, functional disconnectivity of the striatum has been reported in first-episode psychosis, unaffected relatives of patients,^10^ and individuals in an at-risk mental state for psychosis.^11^ Such changes can be reversed as psychotic symptoms resolve.^12, 13^ Existing studies also suggest that pharmacotherapy appears to modulate rsFC patterns.^14, 15^ A recent study found that antipsychotic therapy modulated abnormalities in brain network topology in individuals responding to treatment.^16^ Additionally, functional connectivity in the dorsal attention network^17^ and hippocampus^18^ was changed after 6 weeks of risperidone treatment.

Pattern recognition techniques have been adopted in neuroimaging studies of neuropsychiatric disorders for characterizing brain abnormality at an individual subject level.^19–25^ Successful applications include differentiation between schizophrenia and bipolar disorders^21^ and between schizophrenia patients and healthy controls,^19, 22, 23^ as well as quantification of brain patterns of unaffected family members of schizophrenia patients.^20^ Therefore, the present study adopted multivariate pattern recognition methods ^19, 26^ to characterize brain network patterns of schizophrenia patients and investigate relationship between brain network changes and symptomatic improvements in schizophrenia patients who received treatments of antipsychotics and a combination of antipsychotics and ECT. We also examined predictive power of the brain network patterns of schizophrenia patients for predicting treatment response.

## Results

### Demographics

The demographic and clinical data are summarized in Table 1. No significant difference in age or gender was found between patients with schizophrenia and healthy controls. However, the level of education of these two groups was significantly different. Cognitive test performance for all of the subjects is shown in Table 1. Compared with healthy controls, patients with schizophrenia obtained significantly lower scores on all three cognitive tests (all *p* < 0.05). The average age of onset of schizophrenia was 23.12 ± 5.71 years, and the duration of illness was 6.23 ± 6.23 years.

**Table 1:** Demographic and clinical features of the participants in each group

Characteristic	Schizophrenia patients	Healthy controls	*p*
	*n* = 34	*n* = 34	
	Mean (SD)	Mean (SD)	
Age (years)	29.35 (8.47)	27.71 (5.11)	0.34
Sex (male/female)	10/24	12/22	0.80
Education (years)	13.12 (2.82)	15.03 (4.06)	0.03
Age of onset (years)	23.12 (5.71)	NA	
Duration of illness (years)	6.23 (6.23)	NA	
PANSS score			
Total	80.59 (7.25)	NA	
Positive	26.94 (2.891)	NA	
Negative	16.74 (2.77)	NA	
General	37.00 (4.31)	NA	
Digit span			
Forward	8.48 (1.09)	9.15 (1.31)	0.03
Backward	5.67 (1.93)	7.41 (1.48)	0.00
Symbol-coding	41.36 (13.21)	69.94 (10.52)	0.00
Verbal fluency			
Total	18.15 (6.17)	23.85 (4.19)	0.00
Correct	16.85 (5.61)	22.76 (4.29)	0.00

### Clinical efficacy results

Thirteen patients in the ECT group were treated successfully without adverse effects. On average, 9.10 ± 1.10 ECT sessions were conducted. Sixteen patients underwent MRI scans twice and were treated with antipsychotics only. The repeated-measures analysis of variance (ANOVA) showed that positive and negative syndrome scale (PANSS) total scores (effect of time: *F*_1,27_ = 87.79, *p* < 0.0001; group × time interaction: *F*_1,27_ = 0.65, *p* > 0.05), positive subscale scores (effect of time: *F*_1,27_ = 109.51, *p* < 0.0001; group × time interaction: *F*_1,27_ = 0.41, *p* > 0.05), negative subscale scores (effect of time: *F*_1,27_ = 13.09, *p* < 0.001; group × time interaction: *F*_1,27_ = 0.03, *p* > 0.05), and general psychopathology scale scores (effect of time: *F*_1,27_ = 59.38, *p* < 0.0001; group × time interaction: *F*_1,27_ = 1.26, *p* > 0.05) after treatment in the two groups were significantly lower than prior to treatment. In the ECT group, PANSS total score decreased from 82.43 (SD ± 7.28) to 55.93 (±12.26) after the ECT, corresponding to a significant 31 % (±18) reduction in PANSS total scores (*t* = 6.37, *p* < 0.001). In the MED-only group, PANSS total score decreased from 79.19 (±7.62) to 57.25 (±14.30), corresponding to a significant 27% (±17) reduction (*t* = 5.67, *p* < 0.001). These two groups presented no significant differences in PANSS total scores (effect of group: *F*_1,27_ = 2.67, *p* > 0.05), positive subscale scores (effect of group: *F*_1,27_ = 0.47, *p* > 0.05), negative subscale scores (effect of group: *F*_1,27_ = 0.03, *p* > 0.05), and general psychopathology scale scores (effect of group: *F*_1,27_ = 1.07, *p* > 0.05). The PANSS scores in the two groups are presented in Table 2.

**Table 2:** Clinical symptoms in patients with schizophrenia before and after 6 weeks of ECT or treatment with antipsychotic medication only

Characteristic	ECT group			MED-only group			*F* values (time × group interaction)	*p*
	Before treatment	After 6 weeks	Change (%)	Before treatment	After 6 weeks	Change (%)		
	*n* = 13			*n* = 16				
	Mean (SD)	Mean (SD)		Mean (SD)	Mean (SD)			
PANSS								
Total	82.43 (7.28)	55.93 (12.26)	31 (18)↓	79.19 (7.62)	57.25 (14.30)	27 (17)↓	0.65	0.43
Positive	27.07 (2.62)	14.07 (5.28)	47 (21)↓	27.06(3.32)	15.56 (5.46)	42 (20)↓	0.41	0.53
Negative	16.86 (2.60)	13.50 (3.46)	18 (24)↓	16.56 (3.27)	13.50 (3.65)	16 (22)↓	0.03	0.87
General	38.50 (5.02)	28.36 (4.88)	25 (16)↓	35.75 (3.75)	28.19 (5.99)	21 (14)↓	1.26	0.27
Digit span								
Forward	8.83 (0.94)	8.83 (1.27)	0 (9)	8.19 (1.28)	8.44 (1.46)	3 (9)↑	0.74	0.40
Backward	5.50 (1.51)	5.92 (1.68)	9 (19)↑	6.06 (2.32)	5.69 (1.74)	1 (26)↓	3.05	0.09
Digit symbol coding	41.00 (8.40)	46.50 (8.53)	15 (15)↑	39.38 (16.24)	47.38 (16.22)	25 (24)↑	1.09	0.31
Verbal fluency								
Total	16.17 (6.15)	15.50 (5.40)	6 (47)↓	18.75 (5.42)	18.94 (6.09)	3 (27)↑	0.17	0.69
Correct	14.92 (5.38)	14.75 (4.88)	8 (44)↓	17.69 (5.25)	17.75 (5.57)	2 (24)↑	0.02	0.90

*ECT* electroconvulsive therapy, *MED-only* treatment with antipsychotic medication only, *PANSS* positive and negative syndrome scale; ↑, increase; ↓, decrease

### Cognitive effects

Repeated-measures ANOVA was used to analyze cognitive test scores before and after treatment in the ECT group and the MED-only group. These two treatments had no effect on digit span (forward: effect of time, *F*_1,27_ = 0.74, *p* > 0.05; interaction: *F*_1,27_ = 0.74, *p* > 0.05; backward: effect of time: *F*_1,27_ = 0.01, *p* > 0.05; interaction: *F*_1,27_ = 3.05, *p* > 0.05), verbal fluency total score (effect of treatment time: *F*_1,27_ = 0.05, *p* > 0.05; interaction: *F*_1,27_ = 0.17, *p* > 0.05), or correct score (effect of treatment time: *F*_1,27_ = 0.003, *p* > 0.05; interaction: *F*_1,27_ = 0.016, *p* > 0.05). Although digit symbol scores slightly increased from before treatment to the endpoint of the study (*F*_1,27_ = 31.76, *p* < 0.0001), the group × time interaction was not significant (*F*_1,27_ = 1.09, *p* > 0.05). No significant difference in cognitive performance was found between these two groups (all *p* > 0.05). These results suggested that processing speed was sensitive to the treatments.

### Widespread dysfunctional connectivity patterns in schizophrenia

**Fig. 1:**
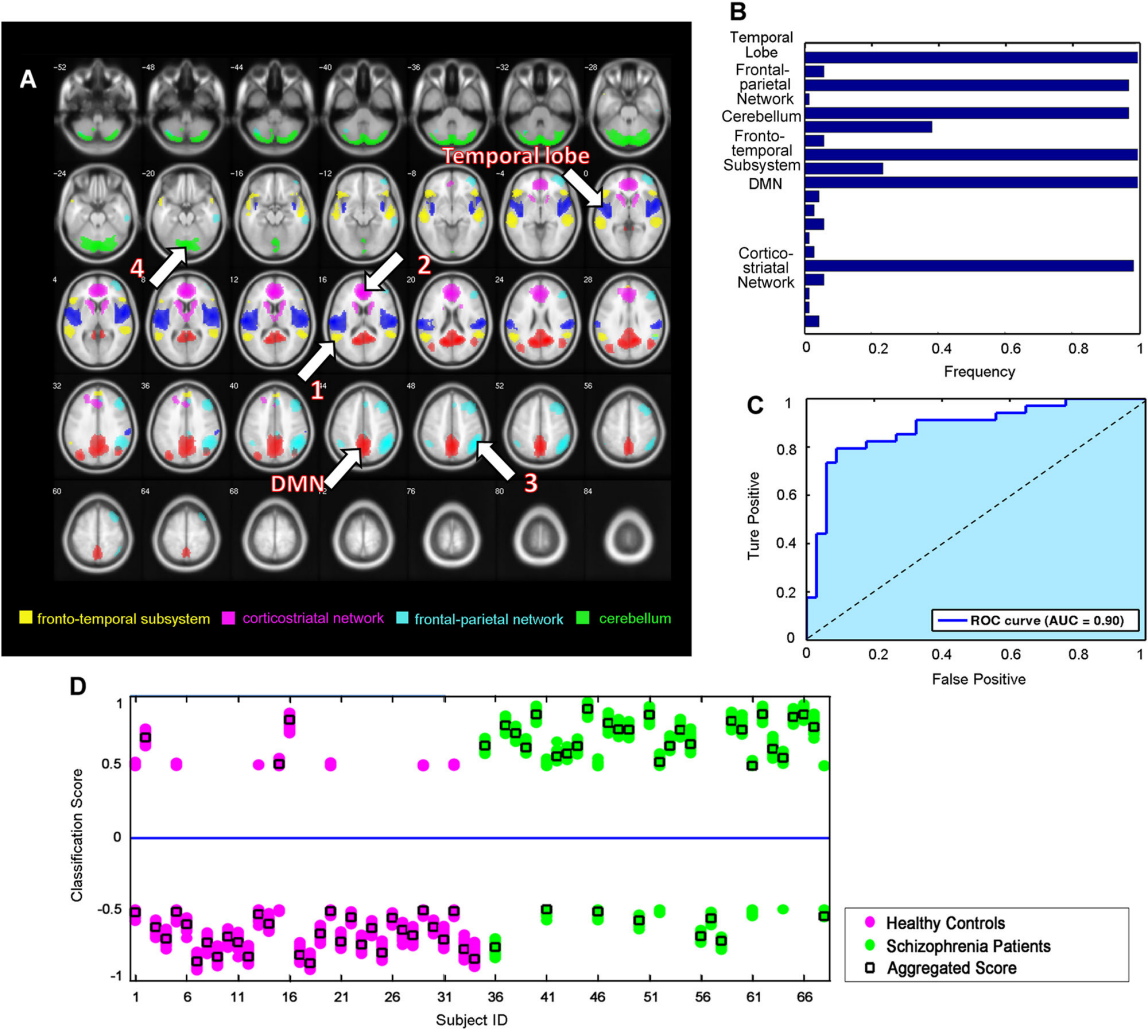
Brain functional connectivity patterns differentiate between schizophrenia patients and healthy controls. **a** Spatial maps of the six selected IC components that could best discriminate between schizophrenia patients and healthy controls. These networks included the language network (fronto-temporal subsystem, *yellow*, IC 13), corticostriatal network (*pink*, IC 5), frontal-parietal network (*bluish green*, IC 18), cerebellum (*green*, IC 16), default mode network (DMN, *red*, IC 11), and temporal lobe network (middle temporal gyrus, *blue*, IC 20). **b** Frequencies of the selected components in leave-one-out mode. Our results showed that the networks that were selected with the highest frequencies (>0.9) were those that constituted the most discriminative functional connectivity pattern. **c** Receiver operating characteristic curve of the most discriminative functional connectivity networks, illustrating good performance of the classifier. **d** Classification scores for each test subject, including scores that were yielded from the base classifiers and aggregated classifiers

Using leave-one-out (LOO) cross-validation, the support vector machine (SVM) classifiers achieved a correct classification rate of 83.82% (specificity = 91.18%, sensitivity = 76.47%, area under the receiver operating characteristic curve [AUC] = 0.90). As shown in Fig. 1, our pattern classification study identified six functional networks that were the most discriminative set for distinguishing schizophrenia patients from healthy controls (91.18% specificity and 76.47% sensitivity). The functional networks were the default mode network (DMN), the temporal lobe network (MTL), the language network (fronto-temporal subsystem), the corticostriatal network, the frontal-parietal network, and the cerebellum (Fig. 1a). Figure 1b shows frequency of the functional networks that were selected in the cross-validation experiments. The receiver operating characteristic curve shown in Fig. 1c indicated that our classifiers might generalize well. The classification results on an independent testing dataset consisting of 20 controls and 33 patients showed that the SVM classifiers achieved a correct classification rate of 75.5% (specificity = 70.00%, sensitivity = 78.79%).

The aggregated and individual base classification scores of testing subjects are shown in Fig. 1d. Compared with healthy controls, schizophrenia patients had significantly higher classification scores. After treatment, the patients’ classification scores were significantly decreased (excluding the misclassified ones, one-tailed permutation test, *n* = 10,000, *p* = 0.028), indicating that effective treatments may drive aberrant disease-related functional networks back to normal.

**Fig. 2:**
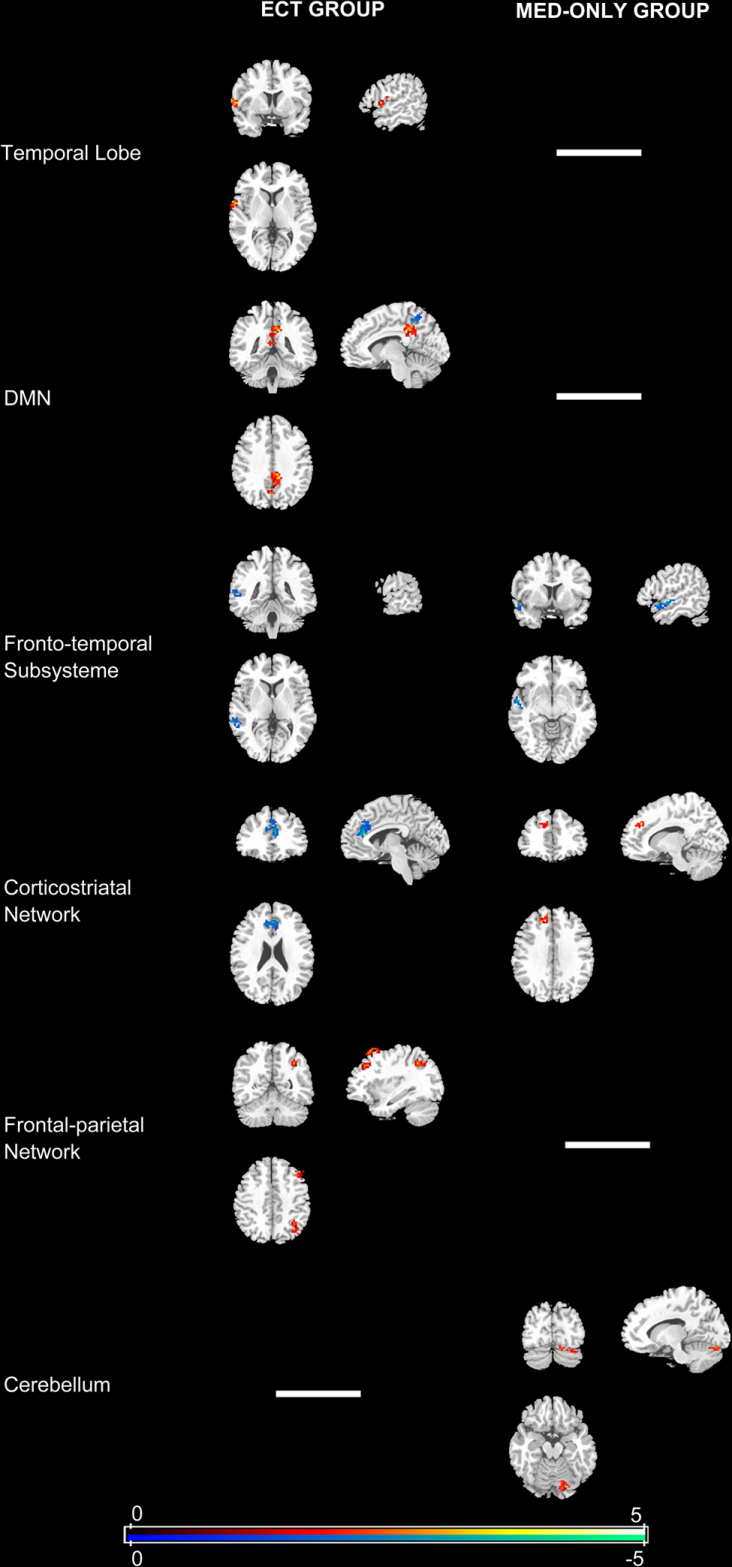
Functional connectivity changes in patients after 6 weeks of treatment compared with baseline (Pseudo paired *t*-test based on permutation test, *n* = 1000, *p* = 0.05). MED-only group: received antipsychotics only; ECT group: ECT plus antipsychotic treatment

We conducted Pseudo paired *t*-test to compare function connectivity of ICNs before and after treatment. After the ECT treatment, the functional connectivity in the DMN (left posterior cingulate cortex, PCC), the temporal lobe network (left superior temporal gyrus) and the frontal-parietal network (right angular gyrus and middle frontal gyrus) increased, meanwhile, the connectivity in the corticostriatal network (right anterior cingulate cortex), the language network (left middle temporal gyrus) and the DMN (right precuneus) decreased. Antipsychotics treatment significantly increased the connectivity in the corticostriatal network (left superior frontal gyrus) and the cerebellum, and decreased the connectivity in language network (left middle temporal gyrus) (Fig. 2).

### Associations between changes in classification scores and clinical response

**Fig. 3:**
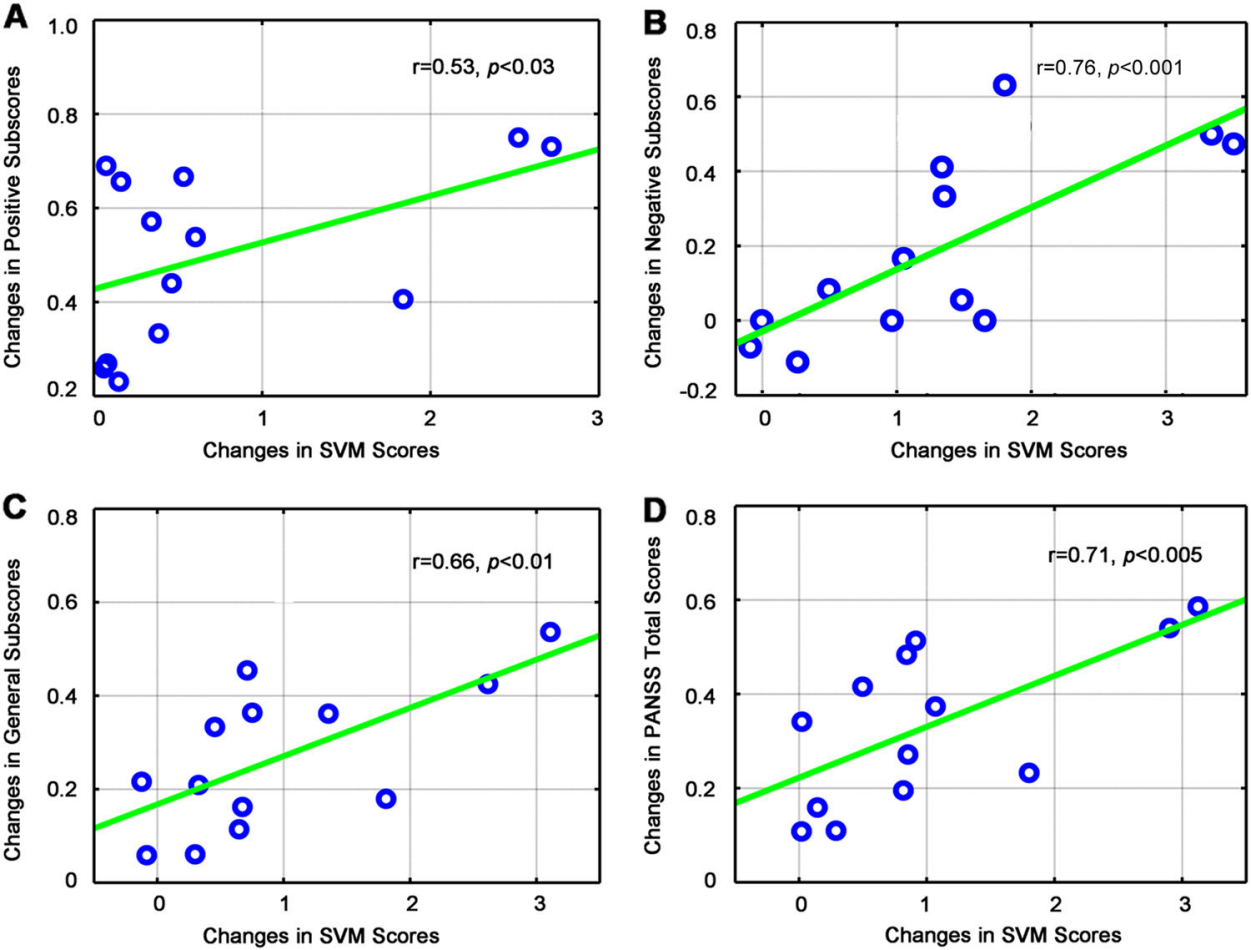
Changes in aggregate scores associated with improvements in psychotic symptoms after electroconvulsive therapy. Scatterplots show associations between changes in SVM scores and symptom improvement as measured by PANSS positive subscores (**a**), PANSS negative subscores (**b**), PANSS general subscores (**c**), and PANSS total scores (**d**). A positive relationship was found between a reduction of psychosis and aggregate scores. This pattern was mirrored by improvements in psychosis, in which these networks showed a significant decrease in functional connectivity. *SVM* support vector machine, *PANSS* positive and negative syndrome scale

A general linear model was built to model relationship between symptom changes and classification score changes for patients who received the treatments. In the ECT group, symptom changes that were measured based on PANSS scores (total score, *r* = 0.71, *p* < 0.005; positive subscore, *r* = 0.53, *p* < 0.03; negative subscore, *r* = 0.76, *p* < 0.001) and general subscores (*r* = 0.66, *p* < 0.01) showed a robust positive correlation with the reduction of classification scores (Fig. 3). However, the correlation between positive subscore and changes in classification scores disappeared after correction for multiple comparisons (*r* = 0.53, *p* < 0.12 Bonferroni corrected). To assess whether extreme outliers in the correlation analyses biased these results, we also calculated all statistics without outliers and obtained similar results (Fig. S1). Multiple linear regression models using least squares were adopted to find outliers whose corresponding residuals were larger than expected in 95% confidence intervals for observations. This pattern indicated that the psychosis changes were significantly correlated with the network changes. However, such correlative patterns were not observed in the MED-only group.

### Predictors of clinical response

**Fig. 4:**
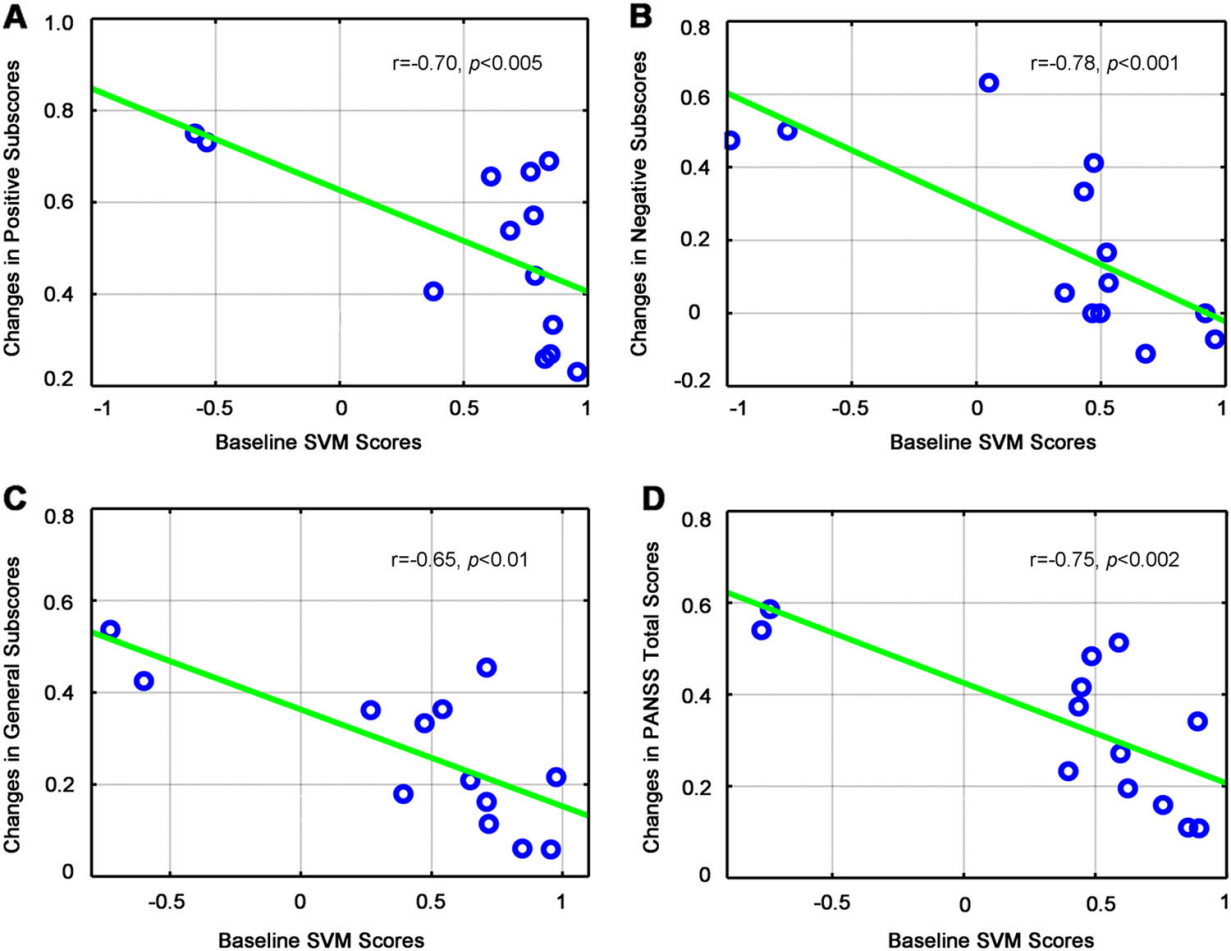
Relationship between baseline classification scores and change in treatment response over the courses of ECT (after controlling for age, gender, years of education, and length of illness). Scatterplots show baseline SVM scores were negatively correlated with the symptom improvement as measured by PANSS positive subscores (**a**), PANSS negative subscores (**b**), PANSS general subscores (**c**), and PANSS total scores (**d**). *ECT* electroconvulsive therapy, *SVM* support vector machine, *PANSS* positive and negative syndrome scale

We established a general linear model to predict the treatment response based on classification scores of the first scan, referred to as baseline classification scores. We found that the treatment response measured based on PANSS scores (total score, *r* = −0.75, *p* < 0.002; positive subscore, *r* = −0.70, *p* < 0.005; negative subscore, *r* = −0.78, *p* < 0.001) and general subscores (*r* = −0.65, *p* < 0.01) was negatively correlated with the baseline classification scores in the ECT group (Fig. 4). Furthermore, these correlations survived Bonferroni correction for multiple comparisons. Patients with lower classification scores of first scan had larger treatment-related changes in PANSS scores. Patients in the MED-only group also presented a similar relationship between changes in PANSS total scores and the baseline classification scores (*r* = −0.49, *p* < 0.03; Fig. S3A). In addition, we performed the correlation analyses without outliers and found that the baseline classification scores significantly correlated with the clinical variables (Fig. S2, S3B).

## Discussion

The present study investigated the effects of ECT on functional networks in a cohort of patients with schizophrenia by combining functional MRI data and machine learning techniques. Resting-state brain functional connectivity in patients differed significantly from healthy controls. The SVM was able to differentiate patients from controls with an accuracy of 83.82% (AUC = 0.90). Together with an improvement of psychosis, we observed a significant decrease in classification scores, suggesting that the functional network patterns in the patient group may shift from a disease state to a healthy state. Lower classification scores before treatment predicted better treatment response. These changes in classification scores were correlated with symptom improvement in the ECT group, whereas this relationship was not found in the MED-only group.

The discriminative brain networks for distinguishing the patients from healthy controls were the DMN, MTL, language network (fronto-temporal subsystem), corticostriatal network, frontal-parietal network, and cerebellum. Functional connectivity and multimodal neuroimaging studies revealed widespread and variable involvement of several brain areas and circuits that contribute to the genesis of psychotic disorders.^27, 28^ For example, auditory hallucinations and disorganized thoughts might result from disruptions of the language network (fronto-temporal subsystem).^29, 30^ Furthermore, Mondino et al.^31^ reported the reduction of auditory verbal hallucinations severity accompanied by decreased rsFC in fronto-temporal subsystem, consistent with our findings. The frontal-parietal network is involved in planning, decision making, and sustained attention, and has been shown to exhibit a reduction of functional connectivity during executive processing in adult schizophrenia patients.^32^ Abnormal white matter integrity of frontal-parietal network in deficit schizophrenia has also been reported as compared with healthy controls.^33, 34^ Although the cerebellum is known to play an important role in the coordination of movement, mounting evidence suggests that the cerebellum is also involved in the processing of emotions and higher cognitive functions.^35, 36^ rsFC studies have demonstrated that the cerebellum is highly interconnected with the DMN and subcortical nuclei and has been shown to exhibit structural and functional abnormalities in schizophrenia.^37^ These disease-related brain disconnections are consistent with existing findings.^22, 38^

The present results showed that both medical treatment and ECT improved the symptom and could drive abnormal brain connectivity patterns back towards normal, thus confirming previous findings in both animal and human studies. Animal studies suggest that the therapeutic efficacy of olanzapine, a commonly prescribed antipsychotic medication, might be mediated by DNA methylation and synaptic plasticity within several brain areas, such as the hippocampus and cerebellum.^39^ We also found increased rsFC in the cerebellum network after 6 weeks antipsychotics treatment. Neuroimaging studies revealed that antipsychotics impact large-scale network organization, and not necessarily specific brain areas.^12, 14, 15^ Elevated striatal activity and a dysfunctional PFC were the most robust abnormalities that were observed in patients with schizophrenia. Antipsychotic treatment has been shown to modulate both striatal metabolism and functional connectivity and to be related to clinical response.^12, 13, 40^ Additionally, studies have also suggested olanzapine appears to modulate the DMN activity, especially enhanced connectivity in the precuneus and reduced connectivity in PCC.^41^ However, no similar connectivity pattern in MED-only group was observed. Furthermore, our analysis found that ECT induced alterations in brain network connectivity patterns. The degree of these changes was related to treatment outcome.

ECT has multiple effects. Many theories of the mechanisms of action of ECT have been proposed, including the enhancement of dopamine synthesis and turnover,^42^ direct effects of convulsions,^43^ and increases in the secretion of BDNF^8, 9^ and neurogenesis.^44^ These hypotheses have gained traction from experimental studies. Several clinical studies have suggested that ECT can serve as an augmentation strategy for clozapine-resistant schizophrenia, which might enhance dopamine D_2_ receptor efficacy.^45^ Moreover, BDNF is involved in the maintenance of midbrain dopaminergic neurons and regulation of synaptic plasticity.^46^ Our findings indicate that the ECT decreases functional connectivity within corticostriatal network. Additionally, seizure activity induced by the ECT may help aberrant cortical excitability return to a balanced state of excitation/inhibition, which would influence the functional connectivity of large scale brain network, thereby alleviating symptoms.

Our results showed that the classification scores of rsfMRI scans before the treatments may be an important predictor of treatment outcome. Patients with lower classification scores before the treatments were closer to the normal state and likely benefited more from the treatments, especially ECT, thus showing improvements in clinical symptoms. However, the relationship between changes in PANSS total score and baseline classification scores was not prominent in the MED-only group. This finding may be explained by the fact that the ECT may induce more pronounced changes in the functional connectivity patterns within a short treatment period, unlike antipsychotic drugs, which may induce subtle changes. Moreover, all of the patients in the ECT group received medication, which might have synergistic effects with ECT. Several neuroimaging studies have sought to discover MRI markers for predicting outcome of the ECT in mood disorders. These studies have generally demonstrated that resting state connectivity in the dorsomedial PFC and anterior cingulate cortex,^47^ intrinsic brain activity in subcallosal cingulate cortex,^48^ and gray matter volume in the amygdala, hippocampus,^49^ and subgenual cingulate^50, 51^ might be associated with individual ECT responses.

The present study has some limitations. First, the patients were not randomized to treatment groups because of the naturalistic study design. However, both treatment groups shared similar clinical features and therefore were comparable. Second, most of the patients had already received antipsychotics before the study. Therefore, no straightforward way is available to compare brain functional alterations between the treatments, which merits further investigation.

In conclusion, the present results suggest that functional connectivity patterns are predictive for therapeutic outcomes in schizophrenia patients, and may help interpret their symptom improvement from a functional connectome perspective. These findings may be helpful for clinicians to identify patients who are most likely to benefit from ECT.

## Materials and methods

### Participants and study design

This study recruited 34 schizophrenia patients and 34 well-matched healthy controls. All of the participants were interviewed using the structured clinical interview for DSM-IV to confirm the diagnosis in patients and rule out current or past psychiatric illness in healthy subjects. The exclusion criteria were the following: (a) <18 or >45 years of age; (b) left handedness; (c) history of brain trauma with loss of consciousness, neurological diseases, or serious physical diseases (respiratory disorders, cardiovascular disease, and so on); (d) diagnosis of alcohol/substance abuse within 12 months before participation; and (e) contraindications for MRI. The study was approved by the Ethics Committee of Beijing Hui-Long-Guan Hospital, Beijing, China, and all of the participants provided written informed consent. Methods were performed in accordance with relevant guidelines and regulations.

At the time of enrollment, all of the patients were experiencing acute exacerbation of psychosis that required hospitalization. Therefore, all of the patients received antipsychotics only (MED-only group) or ECT plus antipsychotic treatment (ECT group) for 6 weeks. These treatment choices were based on clinical decisions and independent from the current study design. A specialized trained psychiatrist (R.J. Zhao) used the PANSS to evaluate psychiatric symptoms in the patients before treatment and at the end of treatment. The treatment response of each patient was measured by changes in its PANSS scores normalized by the baseline scores.

Eight of thirty-four patients during the first scan were free of antipsychotic medication (medication naïve: *n* = 5; off antipsychotic medications for at least 2 weeks: *n* = 3), and all other patients received mono-therapy or dual therapy with antipsychotic medication. In the MED-only group, two patients were treated with first-generation antipsychotic agents alone (haloperidol *n* = 1, sulpiride *n* = 1), eight patients were treated with second-generation antipsychotic agents alone (olanzapine *n* = 3; aripiprazole *n* = 2; paliperidone *n* = 1; risperidone *n* = 1; aripiprazole and olanzapine *n* = 1), two patients were treated with clozapine and a first-generation agent (haloperidol), and four patients were treated with a non-clozapine second-generation and a first-generation agent (risperidone and haloperidol *n* = 2; aripiprazole and haloperidol *n* = 1; olanzapine and chlorpromazine (CPZ) *n* = 1). In the ECT group, nine patients were treated with second-generation antipsychotic agents alone (olanzapine *n* = 4; clozapine *n* = 1; aripiprazole *n* = 1; paliperidone *n* = 1; aripiprazole and risperidone *n* = 2), and four patients were treated with a non-clozapine second-generation and a first-generation agent (risperidone and haloperidol *n* = 2; aripiprazole and haloperidol *n* = 1; olanzapine and CPZ *n* = 1). The doses of antipsychotic medications were converted to CPZ-equivalent doses. No significant differences in antipsychotic doses were found between the ECT (431.67 ± 40.17 mg/day) and MED-only subjects (586.37 ± 57.81 mg/day) (*p* = 0.12). Concomitant benzodiazepines or antidepressant medication was allowed to be used as clinically indicated (lorazepam for five subjects, clonazepam for four, zopiclone for three, estazolam, zaleplon, and citalopram for two subject each, sertraline for one). Patients were also treated with trihexyphenidyl or propranolol as needed for extrapyramidal symptoms. We also applied a battery of cognitive tests to evaluate the cognitive state of the patients. Cognitive ability was examined in the domains of processing speed, attention, and executive function using digit symbol coding, digit span, and verbal fluency tests, respectively. Patients (*n* = 29) with both baseline and follow-up fMRI scans were included in the statistical analysis.

An independent testing data set consisting of 20 controls and 33 schizophrenia patients was used to evaluate the classifiers which were trained using the training data. Demographic and clinical characteristics of the testing data are provided in Supplementary Table 1.

### Electroconvulsive therapy

ECT was performed using an integrated instrument (MECTA spECTrum 5000Q, MECTA Corp, Tualatin, Oregon). Bilateral ECT was applied to patients from 8:30 AM to 9:30 AM. The static resistance was 300–3000 Ω. According to heart rate, the intravenous injected dosages of atropine ranged from 0.25 to 1 mg. The intravenous injected dosages of propofol (anesthetic) and succinylcholine (muscle relaxant) ranged from 1 to 2 mg/kg. After fasciculation disappeared and the muscles relaxed, the patients were given oral tutamen, and the stimulus intensity was adjusted according by an energy percentage based on the patients’ ages. Electrocardiogram, electroencephalogram, electromyography, oxyhemoglobin saturation, and blood pressure were monitored. ECT was applied five times in the first week, three times in the second week, two times in the third week, and taper off. Seven to ten sessions of ECT were given. During ECT, no patient received medication (e.g., lithium, benzodiazepines, and antiepileptic drugs) that could attenuate the therapeutic effect or exaggerate side effects.

### Image acquisition

Structural and resting state fMRI (rsfMRI) scans were collected for all of the subjects, and schizophrenia patients were scanned prior to treatment and after treatment with either antipsychotics (*n* = 16) or ECT plus antipsychotics (*n* = 13). The MRI data were acquired using a 3.0 Tesla Magnetom Trio scanner. The structural scans were acquired using a T1 MPRAGE sequence with the following acquisition parameters: matrix size, 256 × 256; 192 contiguous axial slices; slice thickness, 1 mm; voxel resolution, 1 × 1 × 1 mm^3^; flip angle, 7°; echo time, 3.44 ms; repetition time, 2530 ms; inversion time, 1100 ms. The rsfMRI scans were obtained using a gradient-recalled echo-planar imaging sequence with a repetition time of 2000 ms, echo time of 30 ms, and flip angle of 90°. The slice thickness was 4 mm (no gap) with a matrix size of 64 × 64 and a field of view of 220 × 220 mm^2^, resulting in a voxel size of 3.4 × 3.4 × 4.0 mm^3^. Each rsfMRI brain volume comprised 33 axial slices, and each functional run contained 240 image volumes. During data acquisition, the subjects were instructed to close their eyes, relax, and remain awake. All of the participants were monitored to ensure they were not asleep. All of the images were checked for artifacts, structural abnormalities, and pathologies by a qualified neuroradiologist.

### rsfMRI data preprocessing

Image preprocessing was performed with Statistical Parametric Mapping 8 (SPM8) software (http://www.fil.ion.ucl.ac.uk/spm/software/spm8/), analysis of functional neuroImage (AFNI) software (http://afni.nimh.nih.gov/afni), and the FMRIB Software Library (http://fsl.fmrib.ox.ac.uk/fsl). To allow for magnetization equilibrium, the first six volumes of the functional images were discarded. The preprocessing procedure included slice-timing correction and head motion correction first. Each fMRI scan was intensity-scaled to yield a whole-brain mean value of 10,000. The temporal band-pass filtering (0.01 Hz < f < 0.08 Hz) was performed, and the time series in white matter and cerebrospinal fluid and six affine motion parameters were also regressed out of the data. The removal of linear and quadratic trends was also implemented. The fMRI scans were nonlinearly normalized to Montreal Neurological Institute space with the deformation field obtained with their co-registered T1 scans using DARTEL within SPM8 and resampled to 3 × 3 × 3 mm^3^. Finally, the data were spatially smoothed with a 6 mm full width at half-maximum Gaussian kernel.

### Intrinsic functional networks and pattern classification analysis

Intrinsic connectivity networks (ICNs) were computed for each rsfMRI scan using independent-component analysis (ICA). Group independent components (ICs) were computed using GIFT software (http://mialab.mrn.org/software/gift/), and the number of components was empirically determined to be 20. Group information-guided ICA was used to compute subject-specific ICs^26^ so that each rsfMRI scan could be characterized with 20 subject-specific ICs (Fig. S4). Finally, a pattern classification method was applied to ICs of the baseline data for 34 patients and 34 healthy controls to identify the most discriminative combination of ICs and build SVM classifiers to distinguish patients from controls at an individual subject level with a LOO cross-validation procedure.^19^ Particularly, ICs corresponding to the DMN^52, 53^ and the MTL^54, 55^ were used as a priori knowledge in the pattern classification.^19^ In the LOO cross-validation, one subject was used as a testing sample, and a SVM classifier was built upon the remaining subjects to classify the testing sample. This procedure was repeated until all of the subjects had been used as testing samples. Therefore, classification performance could be estimated based on all of the available subjects. To avoid any bias in the classification, the classifiers were built with a nested LOO procedure based on the training data to optimize their performance by tuning SVM parameters (C and SVM kernel parameters) with grid-searching and choosing the most discriminative combination of ICs with a forward component selection algorithm, yielding a number of SVM classifiers, referred to as base classifiers (*n* = the number of training subjects).^19^ Each of the base classifiers generated a classification score that was a probability value with a sign indicating schizophrenia (positive) or normal (negative),^56^ and the median of base classification scores was used as an aggregated classification score for each testing subject. Each patient’s rsfMRI scan collected after treatment was also classified by the most discriminative SVM classifiers that were built upon the baseline rsfMRI scans while excluding the patient under testing. Therefore, patients with follow-up scans had two classification scores: one for the first scans (baseline classification scores) and the other for scans after treatment (follow-up classification scores).

Voxelwise functional connectivity measures of each ICN were computed as Pearson correlation coefficients between its corresponding time courses and voxelwise fMRI signals and transferred to *z*-scores for every subject. Then, the functional connectivity measures were compared before and after the treatment for the ECT group and MED-only group, respectively. The statistical threshold was Pseudo paired *t*-test based on permutation test, *n* = 1000, *p* = 0.05.

### Correlation analysis between behavioral data and diagnosis scores

To explore the relationships between clinical symptom improvement and changes in functional connection patterns gauged with the classification scores, a general linear model, with age, gender, years of education, and length of illness as covariates, was conducted. Particularly, the classification scores of rsfMRI scans before the treatments, referred to as baseline classification scores, and changes in the classification scores (baseline classification scores minus follow-up classification scores) were correlated with the treatment response. We used Spearman’s rank correlation coefficient (i.e. a nonparametric measure of statistical dependence between two variables) in the correlation analysis.

### Data availability

All the material will be available on request from the corresponding author.

### **CHANGE HISTORY**

A correction to this article has been published and is linked from the HTML version of this article.

## Electronic supplementary material

Supplementary Information
