# Erratum: Electroconvulsive therapy-induced brain functional connectivity predicts therapeutic efficacy in patients with schizophrenia: a multivariate pattern recognition study

**DOI:** 10.1038/s41537-017-0024-6

**Published:** 2017-09-20

**Authors:** Peng Li, Ri-xing Jing, Rong-jiang Zhao, Zeng-bo Ding, Le Shi, Hong-qiang Sun, Xiao Lin, Teng-teng Fan, Wen-tian Dong, Yong Fan, Lin Lu

**Affiliations:** 1 Institute of Mental Health, National Clinical Research Center for Mental Disorders, Key Laboratory of Mental Health and Peking University Sixth Hospital, Peking University, Beijing, 100191 China; 2 National Laboratory of Pattern Recognition, Institute of Automation, Chinese Academy of Sciences, Beijing, 100190 China; 3 University of Chinese Academy of Sciences, Beijing, 100049 China; 4 Department of Alcohol and Drug Dependence, Beijing Hui-Long-Guan Hospital, Peking University, Beijing, 100096 China; 5 National Institute on Drug Dependence and Beijing Key laboratory of Drug Dependence, Peking University, Beijing, 100191 China; 6 Peking-Tsinghua Center for Life Sciences and PKU-IDG/McGovern Institute for Brain Research, Peking University, Beijing, 100871 China; 7 Department of Radiology, Perelman School of Medicine, University of Pennsylvania, Philadelphia, PA 19104 USA

**Erratum to:** *npj Schizophrenia* (2017); doi:10.1038/s41537-017-0023-7; Published 11 May 2017

The Competing interests section states, “The authors declare competing financial interests.” However, it should be, “The authors declare no competing financial interests.” These errors have now been corrected in the HTML and PDF versions of this article.

