# Supplementary material for: Electroconvulsive therapy-induced brain functional connectivity predicts therapeutic efficacy in patients with schizophrenia: a multivariate pattern recognition study
Source: NPJ Schizophr. 2017 May 11;3:21. doi: 10.1038/s41537-017-0023-7 (PMC5441568; doi:10.1038/s41537-017-0023-7)

Supplemental information

Supplementary Tables: 1

Supplementary Figures: 4

Supplementary Tables

Table S1. Demographic and clinical characteristics for the new dataset

Characteristic	Schizophrenia patients<br>*n* = 33<br>Mean (SD)	Healthy controls<br>*n* = 20<br>Mean (SD)	t/X^2	*p*
Age (years)	28.76 (8.91)	24.16 (6.66)	-1.96	0.06
Sex (male/female)	17/16	14/6	1.75	0.19
Education (years)	12.70 (2.78)	11.47 (3.19)	-1.45	0.15
Age of onset (years)	26.44 (9.13)	NA		
Duration of illness (years)	2.61 (2.05)	NA		
PANSS score				
Total	76.78 (6.23)	NA		
Positive	25.63 (2.75)	NA		
Negative	15.93 (2.95)	NA		
General	35.30 (3.92)	NA		

Supplementary Figures

Fig S1: Associations between changes in SVM scores and symptom improvement as measured by PANSS scores without outliers in ECT group. SVM: support vector machine; PANSS: Positive and Negative Syndrome Scale; ECT: electroconvulsive therapy.

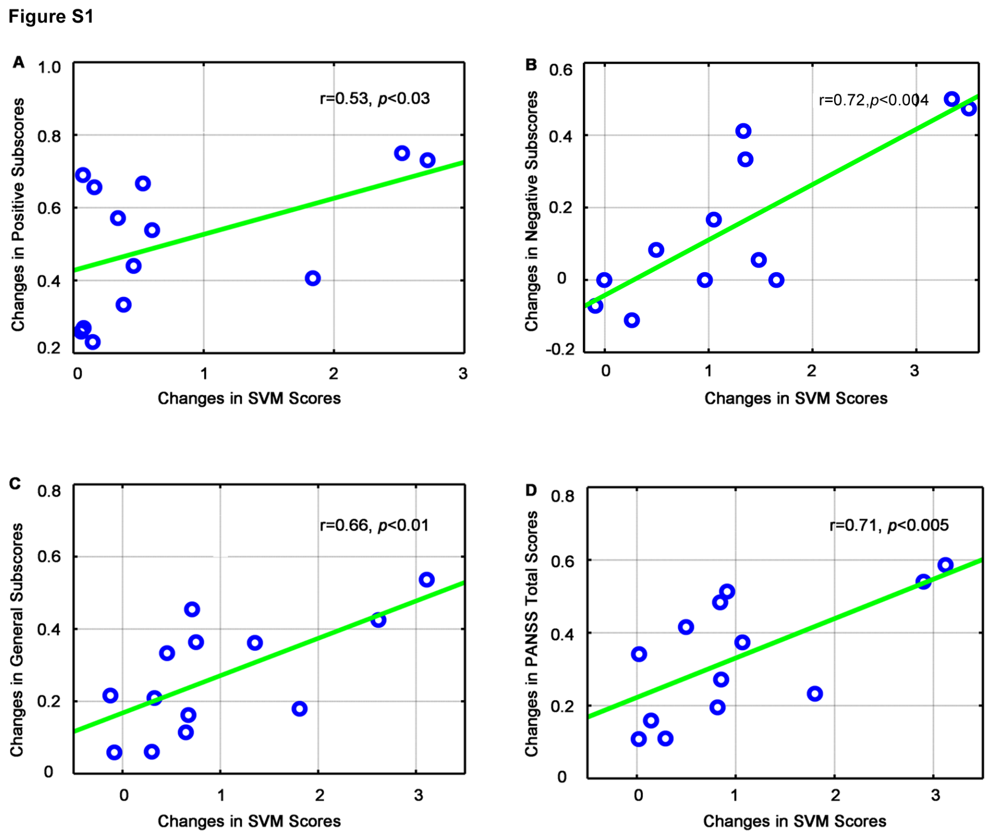

Fig S2: Scatterplots of baseline SVM scores that negatively correlated with the symptom improvement as measured by PANSS scores without outliers in ECT group. SVM: support vector machine; PANSS: Positive and Negative Syndrome Scale; ECT: electroconvulsive therapy.

Figure S2

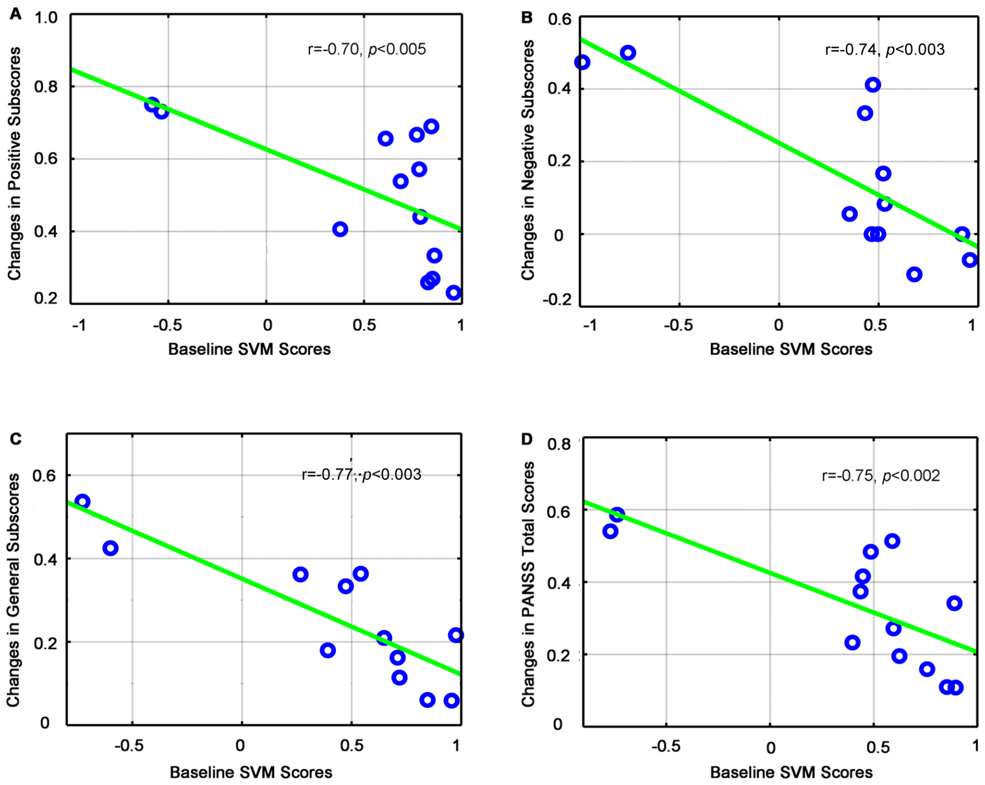

Fig S3: Associations between baseline SVM scores and improvement of clinical symptoms based on the Positive and Negative Syndrome Scale total scores (r=0.49, *p*<0.03) in MED-only group. SVM: support vector machine.

Figure S3

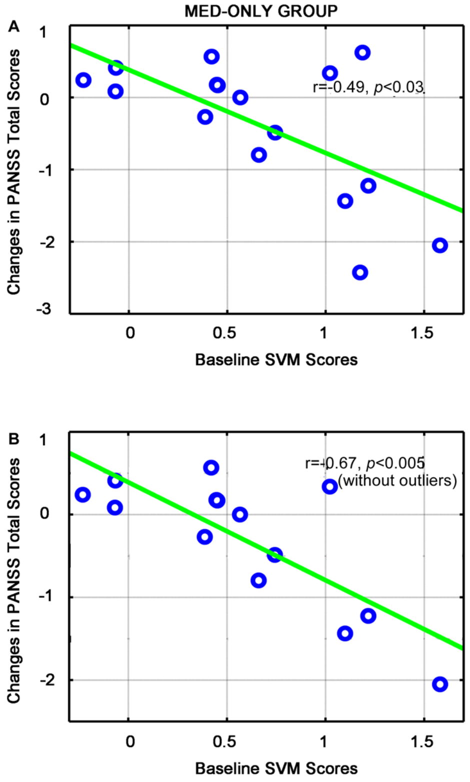

Figure S4. Twenty average independent components (ICs) obtained using GIG-ICA. The sagittal, coronal, and axial views of the 20 mean ICs are displayed in radiological convention (left is right). The z map of each IC was obtained with threshold of p<0.01(FWE corrected) and cluster size ⩾ 50. GIG-ICA: group information-guided independent-component analysis; FWE: family-wise error.

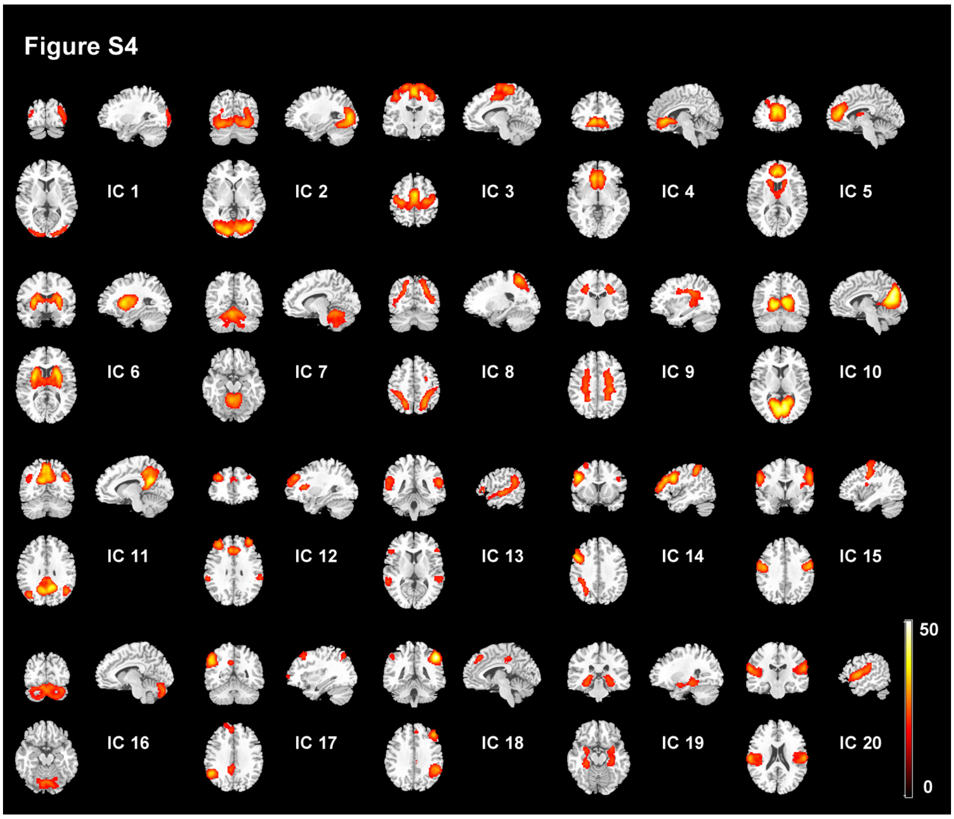

Supplement: Supplementary file 1 — Supplementary Information [file 41537_2017_23_MOESM1_ESM.pdf]
